# Cystic Adenomatoid Odontogenic Tumor

**DOI:** 10.1155/2015/503059

**Published:** 2015-10-22

**Authors:** Sonal Grover, Ahmed Mujib Bangalore Rahim, Nithin Kavassery Parakkat, Shekhar Kapoor, Kumud Mittal, Bhushan Sharma, Anil Bangalore Shivappa

**Affiliations:** ^1^Department of Oral Pathology and Microbiology, Christian Dental College, CMC, Ludhiana, Punjab 141008, India; ^2^Department of Oral Pathology & Microbiology, Bapuji Dental College and Hospital, Davangere, Karnataka 577004, India; ^3^Department of Oral Pathology & Microbiology, Asan Memorial Dental College and Hospital, Keerapakkam Village, Chengalpet, Kanchipuram, Tamil Nadu 603105, India; ^4^Department of Oral Medicine and Radiology, Christian Dental College, CMC, Ludhiana, Punjab 141008, India; ^5^Department of Oral Medicine & Radiology, Sarabha Dental College & Hospital, Ludhiana, Punjab 141105, India; ^6^Department of Oral Pathology & Microbiology, Anil Neerukonda Institute of Dental Sciences, Visakhapatnam, Andhra Pradesh 531162, India

## Abstract

Adenomatoid Odontogenic Tumor (AOT) is a well-established benign epithelial lesion of odontogenic origin. Rightfully called “the master of disguise,” this lesion has been known for its varied clinical and histoarchitectural patterns. Not only does AOT predominantly present radiologically as a unilocular cystic lesion enclosing the unerupted tooth (which is commonly mistaken as a dentigerous cyst) but the lesion also presents rarely with a cystic component histopathologically. We present one such unusual case of cystic AOT associated with an impacted canine, mimicking a dentigerous cyst. The present case aims to highlight the difference between cystic AOT and dentigerous cyst radiographically. The exact histogenesis of AOT and its variants still remains obscure. An attempt has been made to hypothesize the new school of thought regarding the origin of AOT.

## 1. Introduction

Adenomatoid Odontogenic Tumor (AOT) is a well-established benign epithelial lesion of odontogenic origin. The first reported case of AOT which meets the diagnostic criterion was reported by Steensland in 1905 as “epithelioma adamantinum.” In 1971, WHO defined this lesion as, “A tumour of odontogenic epithelium with duct-like structures and with varying degrees of inductive change in the connective tissue. The tumour may be partly cystic, and in some cases the solid lesion may be present only as masses in the wall of a large cyst. It is generally believed that the lesion is not a neoplasm [1, 2].” Though the definition states the lesion may have a cystic nature very few case reports have described the cystic lining. Cystic presentation of AOT has been reported way back in 1915 by Harbitz who reported the lesion as “cystic adamantoma” [3]. In this paper, we report a case of cystic AOT associated with an impacted canine, mimicking a dentigerous cyst. There is a significant radiographic finding evident in this case, which facilitated the exclusion of dentigerous cyst from the final diagnosis. The exact histogenesis of different variants of AOT is open to conjecture. In the present case, we propose Hertwig's Epithelial Root Sheath (HERS) as the source of biphasic (cystic as well as neoplastic) odontogenic epithelium.

## 2. Case Report

**Figure 1:**
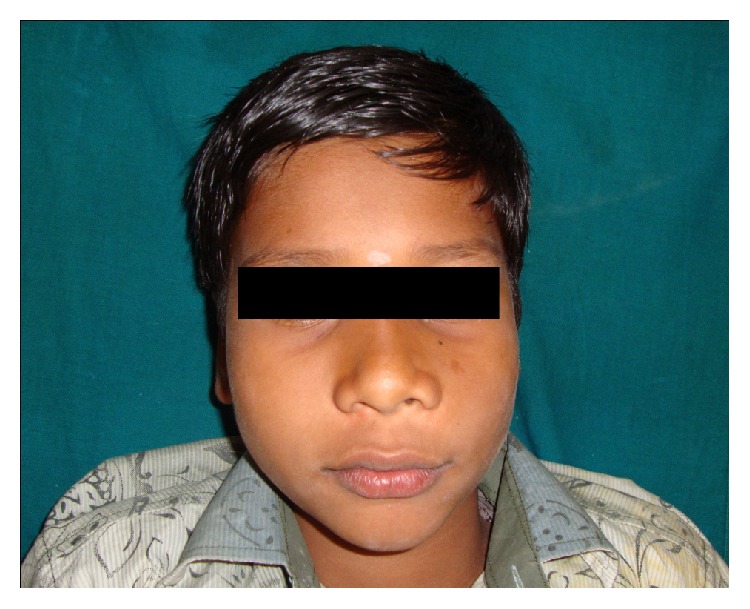
Extra-oral photograph showing sparingly evident swelling in the left middle third of face.

**Figure 2:**
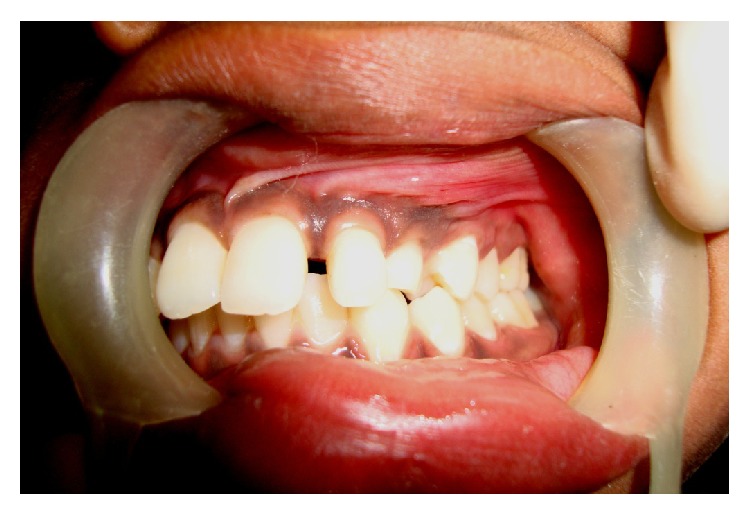
Intraoral photograph showing retained deciduous canine with vestibular obliteration.

**Figure 3:**
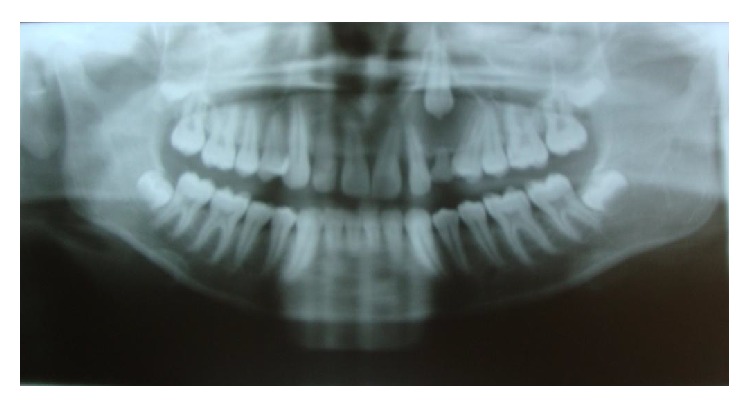
OPG showing impacted maxillary canine with associated radiolucency.

**Figure 4:**
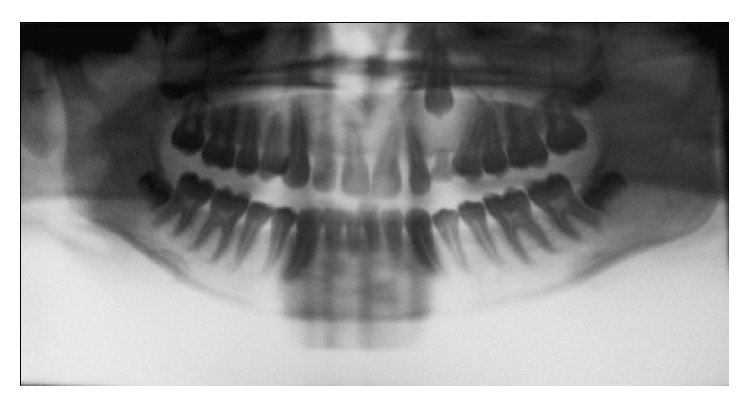
OPG (colors inverted using Photoshop) showing sclerotic margins of the radiolucency enclosing the entire tooth (attached at the apex and not at the cervix of the tooth).

**Figure 5:**
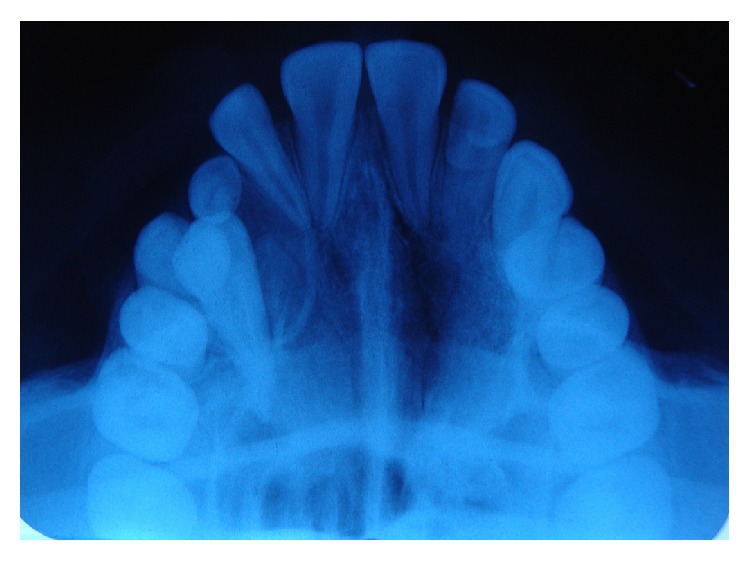
Occlusal radiograph confirming the presence of an impacted maxillary canine.

**Figure 6:**
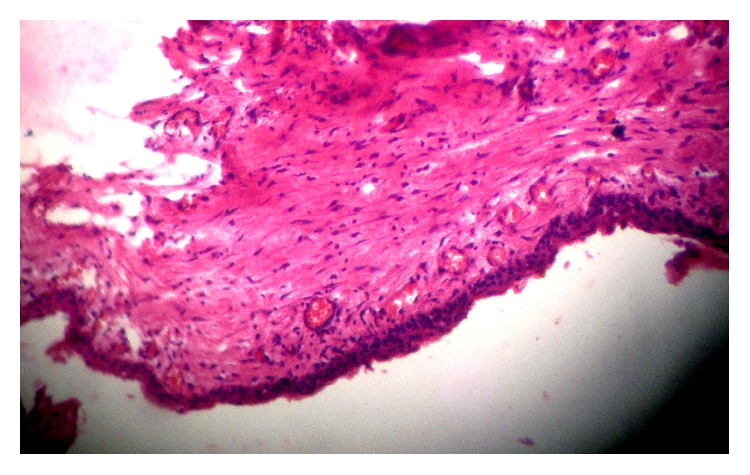
Incisional biopsy showing 2-3 cell layers thick nonkeratinized cystic epithelium, resembling reduced enamel epithelium.

**Figure 7:**
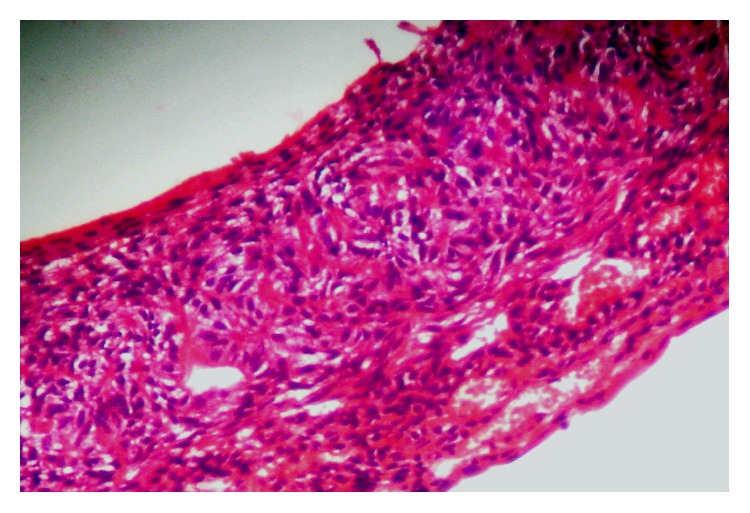
Excisional biopsy showing overlying cystic epithelium with the underlying capsule comprised of cuboidal to columnar epithelial cells forming a rosette-like structures about a central space containing eosinophilic material.

**Figure 8:**
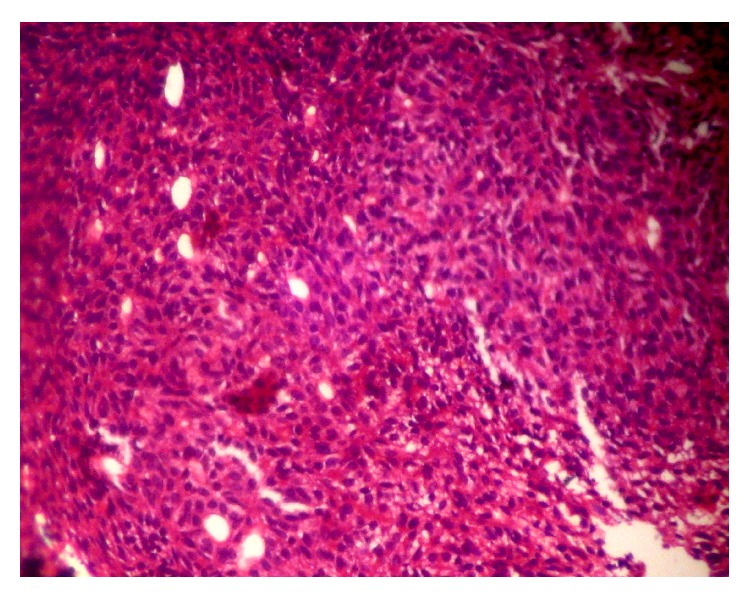
Excisional biopsy showing tubular or duct-like structures.

A 14-year-old boy presented at outpatient department with firm, nontender swelling of left maxillary region, for 6 months. There was no history of trauma. His extra-oral examination showed mild facial asymmetry, with the solitary diffuse swelling sparingly evident in left middle third of face, extending from 1 mm above the ala of the nose till the left commissure (Figure 1). Bilateral submandibular lymph nodes were tender on palpation and were about 2 × 1 cms each. Intra-oral examination revealed the presence of retained deciduous tooth 63. Diffuse swelling measuring 2 × 3 cms in size with vestibular obliteration in relation to 21–24 was evident (Figure 2). A panoramic radiograph revealed a well-defined radiolucency with sclerotic rim enclosing whole of an impacted canine (23) in left maxilla (Figure 3). The sclerotic rim was seen to be attached at the apex and not at cervical region of the tooth (differentiating factor between an AOT and dentigerous cyst) (Figure 4). An occlusal radiograph confirmed the presence of an impacted 23 (Figure 5). On the basis of clinical and radiographic findings, the provisional diagnosis of follicular AOT with an impacted 23 was given. An incisional biopsy was performed and to our surprise, cystic lining comprised of 2-3 cell layers thick, nonkeratinized epithelium resembling reduced enamel epithelium was seen (Figure 6). The mass was enucleated in toto and the cyst was separated out easily from the adjoining bone and was removed with the involved tooth. Histopathological examination of the specimen revealed cystic epithelium, 2-3 layers in thickness, resembling reduced enamel epithelium. Predominantly, the supporting connective tissue capsule was comprised of bundles of collagen fibers, arranged parallel to cystic epithelium. In one bit, overlying cystic epithelium was seen with the underlying capsule comprised of cuboidal to columnar epithelial cells forming a rosette-like structures about a central space containing eosinophilic material (Figure 7). In the same bit, few tubular or duct-like structures were also seen. These ducts consist of central space surrounded by a layer of cuboidal to columnar cells, nuclei of which were polarized away from the central space (Figure 8). Correlation of the histological findings with clinical and radiographic findings persuaded us to give a final diagnosis of cystic AOT. The postoperative course was uneventful and there were no signs of recurrence till 2 years later.

## 3. Discussion

The Adenomatoid Odontogenic Tumor is a benign tumor that is keen to involve the anterior region of the maxillary bones, with a larger number of cases in females, in their second decade of life [1]. It occurs in intraosseous as well as in peripheral forms. Radiographically, the intrabony variants comprise a follicular and an extra-follicular type. The follicular type shows a well-defined, unilocular (round or ovoid) radiolucency associated with the crown and often part of the root of an unerupted tooth thus mimicking a dentigerous cyst. In fact, 77% of follicular type AOTs are initially diagnosed as dentigerous cysts [3]. The characteristic radiographic difference between dentigerous cyst and follicular AOT is that the radiolucency in the former is never associated with part of the root (always attached at the cervix) whereas in the latter it is most commonly associated with the part of the root. In our present case, the sclerotic margin was seen attached at the apex of the tooth (not at the cervix); thus a provisional diagnosis of follicular AOT was given. The histopathological examination revealed features of both dentigerous cyst and AOT. This prompted us to review the scientific literature for such association and explore the histogenesis for the same. After a profound systematic search on PubMed, we found the ten cases, summarized in Table 1.

AOT has been known for its varied histoarchitectural patterns. There has always been a controversy regarding the true nature of this lesion. It was considered to be an hamartoma clinically, due to its limited size and lack of recurrence [16, 17]. The lesional tissue shows a greater departure from the arrangement of the normal odontogenic apparatus than would be expected in a developmental anomaly, as was thought by few authors in its very initial stages of its inception [17]. Authors who consider AOT to be a benign neoplasm believe that the limited size of most cases stems from the fact that they are detected early and removed before the slow-growing tumor reaches a clinically noticeable size. They also point to the considerable size of some reported cases that had gone undetected and untreated for many years, resulting in facial asymmetry and distortion [18–20]. Marx and Stern [16] considered AOT as a cyst and not a tumor and further gave a new terminology for this lesion, “Adenomatoid Odontogenic Cyst (AOC).” According to them, the AOC does not arise from the follicle of the tooth crown but instead arises from HERS, which would explain the finding of the tooth being completely within the lumen rather than the tooth root being within a bony crypt (as also seen in our present case). Philipsen et al. [1, 21] have strongly argued in favour of the concept of AOT being derived from the complex system of dental laminae or its remnants.

Considering all the case reports of AOT associated with dentigerous cyst, it is very much evident that the tumor is originating from the cystic lining. Moreover, the WHO definition of AOT given by Philipsen et al. supports this fact of cystic lining to be the progenitor for tumor in many cases. Since the origin of the cystic lining in dentigerous cyst is reduced enamel epithelium (REE) and not the dental lamina, we propose the former to be the progenitor of AOT. Immunohistochemically, it has been shown that the immunophenotypic profile of REE and AOT epithelium is virtually identical [22]. In cases, where the tooth is found completely within the lumen (as seen in our present case), we concur with Marx and Stern's opinion of AOT stemming from HERS. We believe all AOTs begin as a cyst derived from either REE or HERS. In classical AOT (solid), the proliferation of nodules originating from the cystic lining fills up the entire lumen whereas in cystic variant this process is incomplete and is thus seen only in parts of cystic lining. Furthermore we consider extra-follicular as well as peripheral AOTs originate from the remnants of HERS (epithelial rests of Malassez), which complies with the common histology for all these variants.

## Figures and Tables

**Table 1:** Clinical and radiographic data of the reported cases of Adenomatoid Odontogenic Tumor associated with a dentigerous cyst.

Authors	Age/sex	Site	Race	Radiographic findings	Other findings
Valderrama [4]	16/female	Maxilla	Filipino	Unilocular radiolucency, surrounding tooth 14	Presence of complex odontoma

Warter et al. [5]	8/male	Maxilla	Nigerian	Unilocular radiolucency, surrounding tooth 13	Contained melanocytes and melanin-laden epithelial cells

Tajima et al. [6]	15/male	Maxillary sinus	Japanese	A well-defined radiopaque mass and crown of unerupted 28	—

Garcia-Pola Vallejo et al. [7]	12/male	Maxilla	Spanish	Unilocular radiolucency, surrounding tooth 23	Agenesis of tooth 15 and 24 crown

Takahashi et al. [8]	22/male	Maxilla	Japanese	Unilocular radiolucency, surrounding tooth 28	Lesion expanding to sinus

Bravo et al. [9]	14/male	Maxilla	Not stated	Unilocular radiolucency, surrounding tooth 23	Lesion expanding to sinus

Nonaka et al. [10]	13/female	Maxilla	Brazilian	Unilocular radiolucency with few radiopaque areas in relation to 23 and 24	—

Chen et al. [11]	15/male	Maxilla	Chinese	Radiolucency around upper deciduous canine	Odontoma-like areas were also observed

Sandhu et al. [12]	25/female	Maxillary sinus	Indian	Unilocular radiolucency, surrounding tooth 13	Cribriform area showing chords of cells surrounding loose edematous stroma was seen

Gadewar and Srikant [13]	12/male	Maxilla	Indian	Unilocular radiolucency, surrounding tooth 13	Erosion of right lateral nasal bone

Agarwal et al. [14]	15/female	Maxilla	Indian	Unilocular radiolucency, surrounding tooth 23	Root resorption of adjacent teeth

Kurra et al. [15]	19/female	Mandible	Indian	Large radiolucency in relation to 37 and 38. Impacted 38	Root resorption of 36
